# Supplementary material for: The relationship between social support, stressful events, and menopause symptoms
Source: PLoS One. 2021 Jan 27;16(1):e0245444. doi: 10.1371/journal.pone.0245444 (PMC7840006; doi:10.1371/journal.pone.0245444)
Supplement: S1 Text — Supporting information containing information on the selection of the social support variable (Figure A), and the derivation of the stress index (Table A) and stress dose (Table B). Figure B demonstrates the justification for how missing data in the stress dose variable was dealt with, and Figure C shows the distribution of the stress dose variable by wave and age. Complete model results are presented in Table C. (DOCX) [file pone.0245444.s001.docx]

# S1 Text. Supporting text.

## Distribution of social support


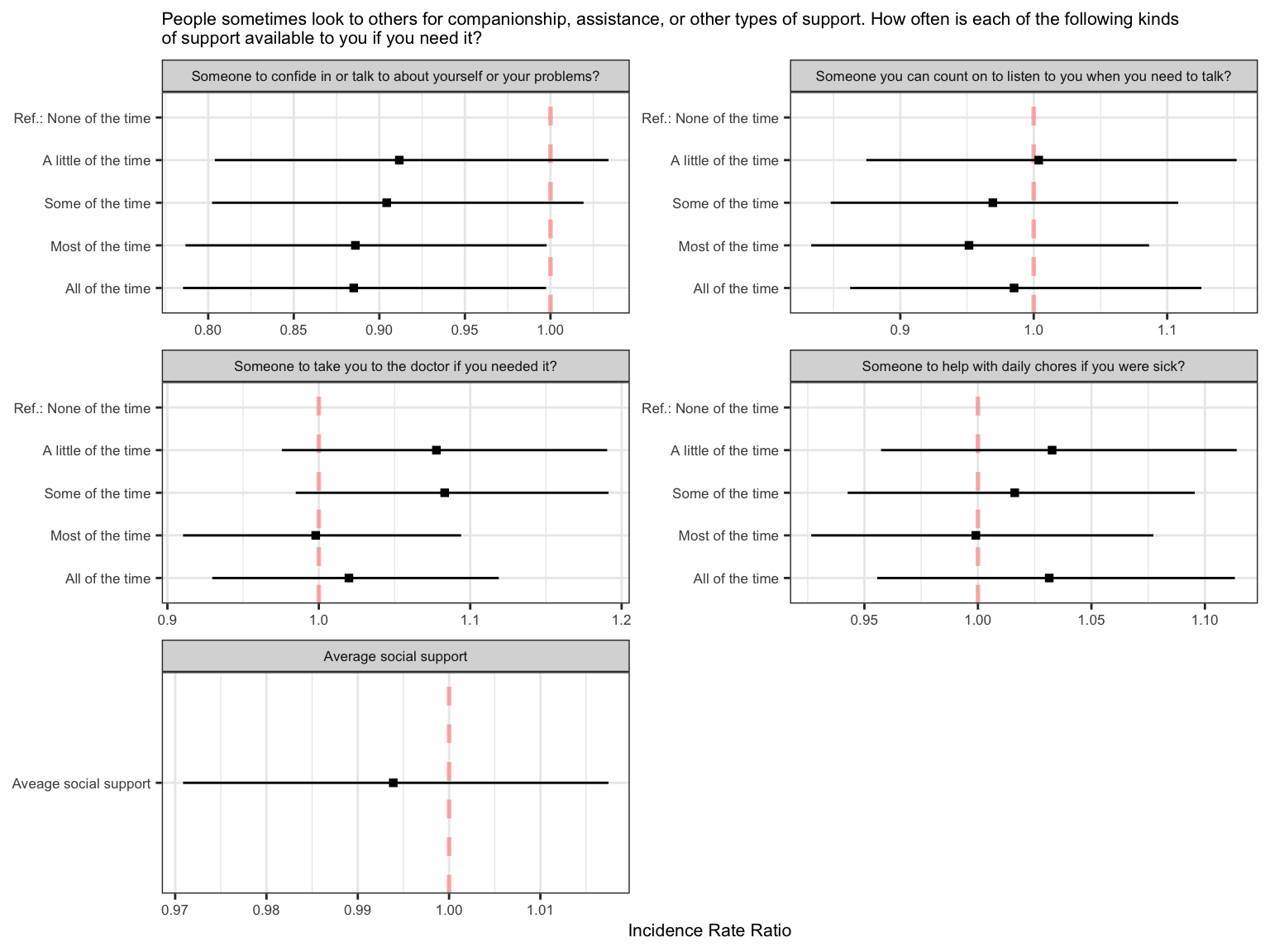


**Figure A. Individual measures of social support.**


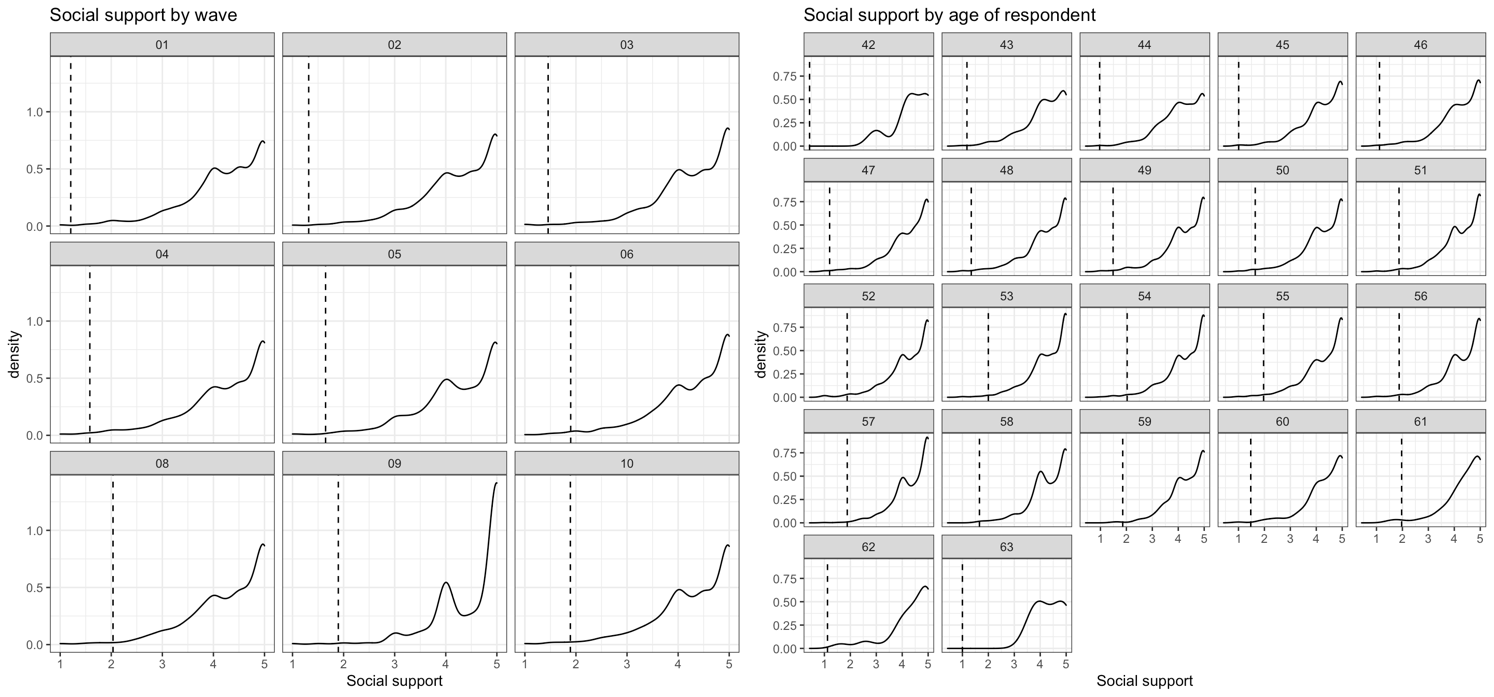


**Figure B. Distribution of the social support variable used in the main analyses by wave and age.** The mean is shown with a dashed line.

## Selection of the stress variable

SWAN collect data on 18 stressors. Each of these was modelled individually against VMS to assess their relationship with such (results from this analysis are presented in the main manuscript). As they all presented a similar relationship, a collapsed measure – the *Stress index* – was created. This involved taking the woman’s maximum experience of stress. For example, if a woman had not experienced any of the stressors, then her stress index would be “No”. However, if she had experienced three of the stressors, with responses of “Yes - not upsetting”, “Yes - not upsetting”, and “Yes - very upsetting”, then her stress index would be “Yes - very upsetting” as that is the maximum amount of stress she experienced (see Table A for a hypothetical derivation of this variable).

| **Table A. Derivation of the *Stress index* variable using hypothetical data** | | | | |
| --- | --- | --- | --- | --- |
| **Person ID** | **Stressor 1** | **Stressor 2** | **Stressor 3** | **Derived stress index** |
| 1 | Yes – not upsetting | Yes – not upsetting | Yes – very upsetting | Yes – very upsetting |
| 2 | No | No | Yes – not upsetting | Yes – not upsetting |
| 3 | Yes – very upsetting and still upsetting | Yes – very upsetting | No | Yes – very upsetting and still upsetting |
| 4 | No | No | No | No |
| 5 | Yes – not upsetting | Yes – somewhat upsetting | Yes – not upsetting | Yes – somewhat upsetting |

In addition to the above *Stress index* variable, we also created a variable to measure a ‘dosage effect’, or the amount of stress experienced. In other words, we counted the number of stressful events the woman reported having experienced, with anything other than “No” being counted. If the data was missing, this was also counted as having not experienced it (see Table B for a hypothetical derivation of this variable). This decision about missing data was made to avoid losing information, as if women with missing data for any of the stressful events was ignored, 127 women would have been dropped from the analysis. To justify this decision in regards to missing data, we present the results from the stress dose including missing women to the stress dose excluding missing women (Figure B), demonstrating that the results are comparable regardless of how missing data are handled.

| **Table B. Derivation of the *Stress dose* variable using hypothetical data.** Responses in red indicate what was counted towards the stress dose. | | | | | | |
| --- | --- | --- | --- | --- | --- | --- |
| **Person ID** | **Stressor 1** | **Stressor 2** | **Stressor 3** | **Stressor 4** | **Stressor 5** | **Derived stress dose** |
| 6 | Yes – not upsetting | Yes – not upsetting | Yes – very upsetting | Yes – somewhat upsetting | Yes – very upsetting | 5 |
| 7 | No | NA | Yes – not upsetting | No | Yes – not upsetting | 2 |
| 8 | No | Yes – very upsetting | No | No | No | 1 |
| 9 | No | No | No | NA | NA | 0 |
| 10 | Yes – not upsetting | NA | NA | No | Yes – very upsetting | 2 |

**Figure D. Distribution of the stress dose variable used in the main analyses by wave and age.** The mean is shown with a dashed line.


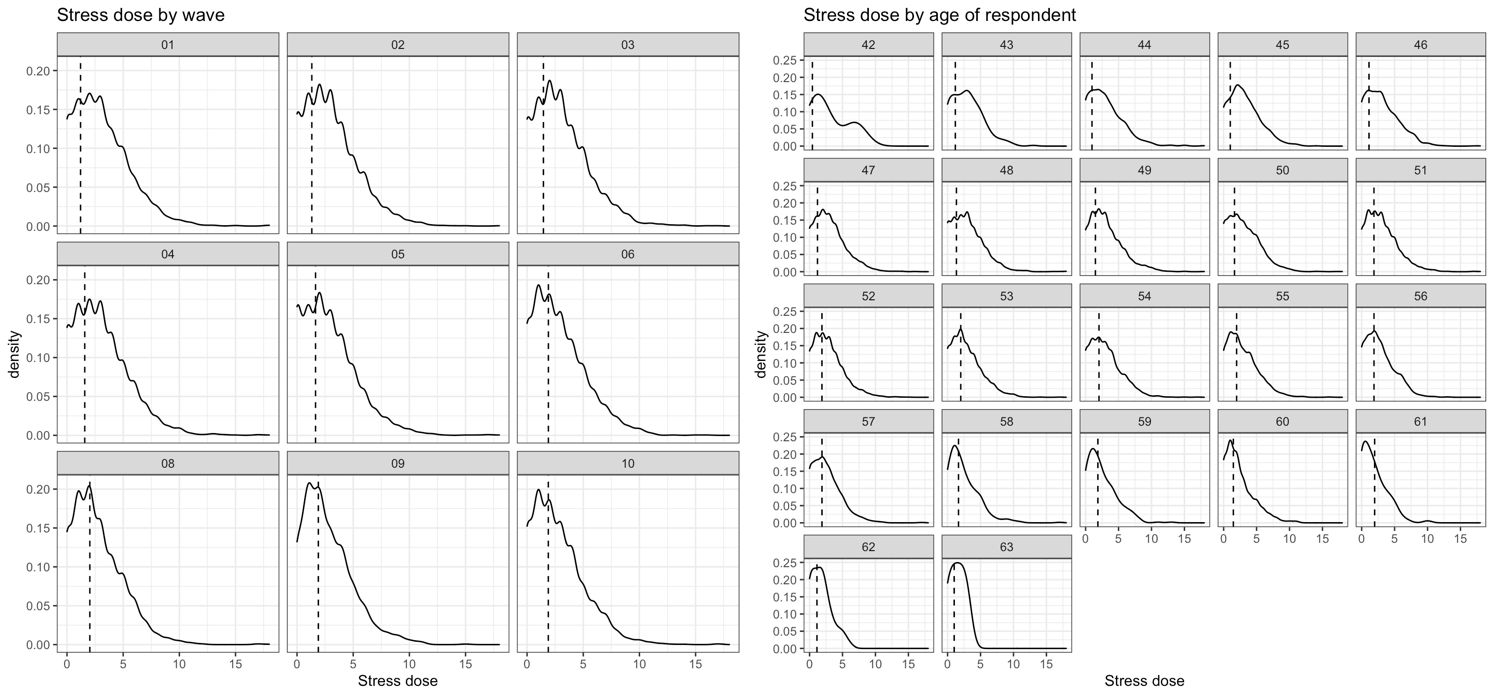


**Figure C. Relationship between the different stress measures and vasomotor symptoms, modelled using multilevel Poisson regression with random effects.** Models are unadjusted, with a higher Incidence Rate Ratio indicating more frequent symptoms.


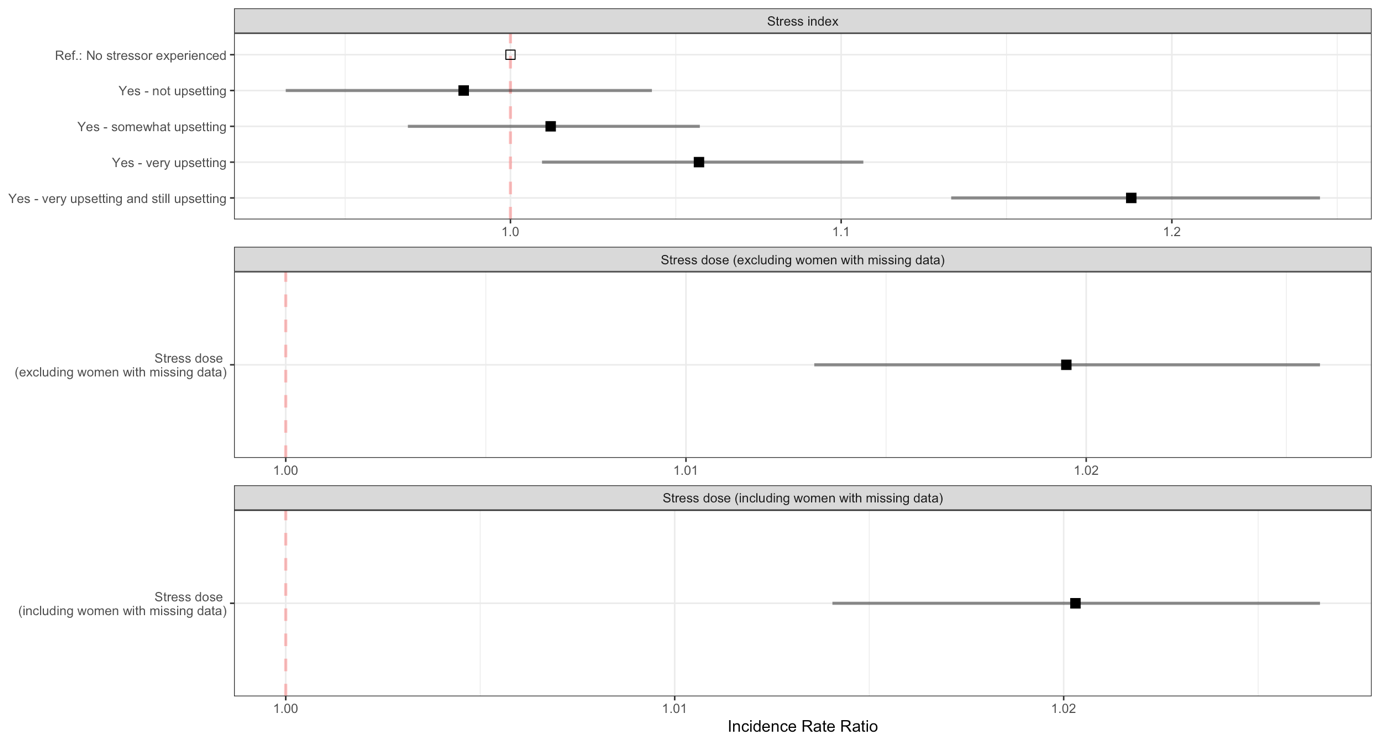


## Full model results

| **Table C. Full model results, reporting Incidence Rate Ratio and 95% confidence intervals.** In the *Lagged model*, the stress index is measured at t-1. | | | | | | | | | |
| --- | --- | --- | --- | --- | --- | --- | --- | --- | --- |
|  | **Dependent variable = vasomotor symptoms** | | | | | | | | |
|  | **Base model** | **Support model** | **Stress model 1** | **Stress model 2** | **Full model 1** | **Full model 2** | **Interaction model 1** | **Interaction model 2** | **Lagged model** |
| Average support received |  | 0.98 (0.96-1.00) |  |  | 0.99 (0.96-1.01) | 0.99 (0.96-1.01) | 1.02 (0.97-1.07) | 1.00 (0.97-1.03) |  |
| Stress index (ref.: No) |  |  |  |  |  |  |  |  |  |
| Yes - not upsetting |  |  | 1.01 (0.95-1.06) |  | 1.01 (0.95-1.06) |  | 1.11 (0.78-1.60) |  | 1.02 (0.96-1.08) |
| Yes - somewhat upsetting |  |  | 1.03 (0.98-1.07) |  | 1.03 (0.98-1.07) |  | 1.02 (0.79-1.30) |  | 1.01 (0.97-1.06) |
| Yes - very upsetting |  |  | 1.07** (1.03-1.13) |  | 1.07** (1.03-1.13) |  | 1.31* (1.01-1.69) |  | 1.00 (0.95-1.05) |
| Yes - very upsetting and still upsetting |  |  | 1.21*** (1.15-1.26) |  | 1.20*** (1.15-1.26) |  | 1.57*** (1.23-2.00) |  | 1.07* (1.01-1.12) |
| Stress dose |  |  |  | 1.03*** (1.02-1.03) |  | 1.03*** (1.02-1.03) |  | 1.05*** (1.02-1.07) |  |
| Age | 1.12*** (1.09-1.15) | 1.12*** (1.09-1.16) | 1.12*** (1.09-1.16) | 1.12*** (1.09-1.16) | 1.12*** (1.09-1.16) | 1.13*** (1.09-1.16) | 1.12*** (1.09-1.16) | 1.12*** (1.09-1.16) | 1.08*** (1.05-1.12) |
| Age^2^ | 0.92*** (0.91-0.93) | 0.92*** (0.91-0.93) | 0.92*** (0.91-0.94) | 0.92*** (0.91-0.94) | 0.92*** (0.91-0.94) | 0.92*** (0.91-0.94) | 0.92*** (0.91-0.94) | 0.92*** (0.91-0.93) | 0.90*** (0.88-0.91) |
| Age^3^ | 0.99* (0.98-1.00) | 0.99* (0.98-1.00) | 0.99* (0.98-1.00) | 0.99* (0.98-1.00) | 0.99* (0.98-1.00) | 0.99* (0.98-1.00) | 0.99* (0.98-1.00) | 0.99* (0.98-1.00) | 1.01 (1.00-1.02) |
| Marital status (ref.: Divorced/Separated/Single) |  |  |  |  |  |  |  |  |  |
| Married/In a relationship | 1.10*** (1.05-1.16) | 1.11*** (1.06-1.17) | 1.11*** (1.06-1.17) | 1.11*** (1.06-1.17) | 1.11*** (1.06-1.17) | 1.11*** (1.06-1.17) | 1.11*** (1.06-1.17) | 1.11*** (1.06-1.17) | 1.10*** (1.04-1.16) |
| Widowed | 1.07 (0.95-1.19) | 1.07 (0.95-1.20) | 1.05 (0.94-1.18) | 1.06 (0.95-1.19) | 1.06 (0.94-1.18) | 1.07 (0.95-1.19) | 1.06 (0.94-1.18) | 1.07 (0.95-1.19) | 1.04 (0.92-1.18) |
| Smoking (ref.: Never smoked) |  |  |  |  |  |  |  |  |  |
| Ever smoked | 1.22*** (1.12-1.32) | 1.22*** (1.12-1.32) | 1.21*** (1.11-1.31) | 1.21*** (1.12-1.31) | 1.21*** (1.11-1.31) | 1.21*** (1.12-1.31) | 1.21*** (1.12-1.31) | 1.21*** (1.12-1.31) | 1.19*** (1.09-1.30) |
| Education (ref.: Less than high school) |  |  |  |  |  |  |  |  |  |
| High school | 0.95 (0.78-1.15) | 0.95 (0.78-1.16) | 0.94 (0.77-1.15) | 0.94 (0.77-1.14) | 0.94 (0.78-1.15) | 0.94 (0.77-1.14) | 0.94 (0.77-1.15) | 0.94 (0.77-1.14) | 0.96 (0.77-1.18) |
| Some college/technical school | 0.92 (0.76-1.12) | 0.92 (0.76-1.12) | 0.91 (0.75-1.10) | 0.90 (0.75-1.09) | 0.91 (0.76-1.11) | 0.91 (0.75-1.10) | 0.91 (0.76-1.11) | 0.91 (0.75-1.10) | 0.94 (0.77-1.15) |
| College degree | 0.70*** (0.57-0.85) | 0.70*** (0.57-0.86) | 0.69*** (0.56-0.84) | 0.69*** (0.56-0.84) | 0.69*** (0.56-0.84) | 0.69*** (0.56-0.84) | 0.69*** (0.56-0.84) | 0.69*** (0.56-0.84) | 0.73** (0.59-0.90) |
| Post-graduate education | 0.72** (0.59-0.88) | 0.72** (0.59-0.88) | 0.71*** (0.58-0.87) | 0.71*** (0.58-0.86) | 0.71*** (0.58-0.87) | 0.71*** (0.58-0.87) | 0.71*** (0.58-0.87) | 0.71*** (0.58-0.87) | 0.74** (0.60-0.92) |
| Ethnicity (ref.: Black/African American) |  |  |  |  |  |  |  |  |  |
| Chinese | 0.44*** (0.37-0.52) | 0.44*** (0.37-0.52) | 0.45*** (0.38-0.54) | 0.45*** (0.38-0.53) | 0.45*** (0.38-0.54) | 0.45*** (0.38-0.53) | 0.46*** (0.39-0.54) | 0.45*** (0.38-0.53) | 0.44*** (0.37-0.52) |
| Japanese | 0.41*** (0.35-0.48) | 0.41*** (0.35-0.48) | 0.42*** (0.36-0.49) | 0.42*** (0.36-0.50) | 0.42*** (0.36-0.49) | 0.43*** (0.36-0.50) | 0.42*** (0.36-0.49) | 0.43*** (0.36-0.50) | 0.43*** (0.36-0.50) |
| Caucasian | 0.70*** (0.64-0.77) | 0.70*** (0.64-0.77) | 0.70*** (0.64-0.77) | 0.71*** (0.65-0.78) | 0.70*** (0.64-0.77) | 0.71*** (0.65-0.78) | 0.70*** (0.64-0.77) | 0.71*** (0.65-0.78) | 0.70*** (0.63-0.77) |
| Hispanic | 0.62*** (0.51-0.75) | 0.62*** (0.51-0.75) | 0.64*** (0.53-0.77) | 0.65*** (0.54-0.79) | 0.64*** (0.53-0.77) | 0.65*** (0.54-0.79) | 0.64*** (0.53-0.77) | 0.65*** (0.54-0.79) | 0.58*** (0.47-0.72) |
| Health (ref.: Poor) |  |  |  |  |  |  |  |  |  |
| Fair | 0.96 (0.88-1.04) | 0.96 (0.88-1.04) | 0.98 (0.90-1.06) | 0.96 (0.88-1.05) | 0.98 (0.90-1.06) | 0.96 (0.88-1.05) | 0.98 (0.90-1.06) | 0.96 (0.88-1.04) | 0.95 (0.87-1.05) |
| Good | 0.82*** (0.75-0.90) | 0.82*** (0.75-0.90) | 0.84*** (0.77-0.92) | 0.83*** (0.76-0.90) | 0.84*** (0.77-0.92) | 0.83*** (0.76-0.90) | 0.84*** (0.77-0.92) | 0.82*** (0.75-0.90) | 0.81*** (0.73-0.90) |
| Very good | 0.74*** (0.67-0.81) | 0.74*** (0.67-0.81) | 0.76*** (0.70-0.84) | 0.75*** (0.68-0.82) | 0.76*** (0.70-0.84) | 0.75*** (0.68-0.82) | 0.77*** (0.70-0.84) | 0.75*** (0.68-0.82) | 0.73*** (0.66-0.82) |
| Excellent | 0.65*** (0.58-0.72) | 0.65*** (0.58-0.72) | 0.67*** (0.60-0.74) | 0.66*** (0.59-0.73) | 0.67*** (0.60-0.74) | 0.66*** (0.59-0.73) | 0.67*** (0.61-0.74) | 0.66*** (0.59-0.73) | 0.64*** (0.57-0.71) |
| Menopausal status (ref.: Early peri) |  |  |  |  |  |  |  |  |  |
| Pre-menopausal | 0.66*** (0.61-0.71) | 0.66*** (0.61-0.71) | 0.66*** (0.61-0.71) | 0.66*** (0.61-0.71) | 0.66*** (0.61-0.71) | 0.66*** (0.61-0.71) | 0.66*** (0.61-0.71) | 0.66*** (0.61-0.71) | 0.66*** (0.60-0.73) |
| Late peri | 1.87*** (1.79-1.96) | 1.87*** (1.79-1.96) | 1.88*** (1.80-1.97) | 1.88*** (1.80-1.97) | 1.88*** (1.80-1.97) | 1.88*** (1.80-1.97) | 1.88*** (1.80-1.97) | 1.89*** (1.80-1.97) | 1.95*** (1.86-2.05) |
| Menopaused | 1.44*** (1.37-1.51) | 1.44*** (1.37-1.51) | 1.44*** (1.37-1.50) | 1.45*** (1.38-1.52) | 1.44*** (1.37-1.51) | 1.45*** (1.38-1.52) | 1.44*** (1.37-1.51) | 1.45*** (1.38-1.52) | 1.52*** (1.45-1.60) |
| Other | 1.07** (1.02-1.13) | 1.07** (1.02-1.13) | 1.07** (1.02-1.13) | 1.08** (1.03-1.13) | 1.07** (1.02-1.13) | 1.08** (1.03-1.13) | 1.07** (1.02-1.13) | 1.08** (1.03-1.13) | 1.11*** (1.05-1.18) |
| Social support:Stress index (ref.: No) |  |  |  |  |  |  |  |  |  |
| Social support:Yes - not upsetting |  |  |  |  |  |  | 0.98 (0.90-1.06) |  |  |
| Social support:Yes - somewhat upsetting |  |  |  |  |  |  | 1.00 (0.95-1.06) |  |  |
| Social support:Yes - very upsetting |  |  |  |  |  |  | 0.96 (0.90-1.01) |  |  |
| Social support:Yes - very upsetting and still upsetting |  |  |  |  |  |  | 0.94* (0.89-0.99) |  |  |
| Social support:Stress dose |  |  |  |  |  |  |  | 1.00 (0.99-1.00) |  |
| Note: * P<0.05; ** P<0.01; *** P<0.001 | | | | | | | | | |
| Age centered in analyses |  |  |  |  |  |  |  |  |  |
