# Supplementary material for: The relationship between social support, stressful events, and menopause symptoms
Source: PLoS One. 2021 Jan 27;16(1):e0245444. doi: 10.1371/journal.pone.0245444 (PMC7840006; doi:10.1371/journal.pone.0245444)
Supplement: Attachment — Submitted filename: MR_Arnot et al. 2020_Reviewer reply.docx [file pone.0245444.s002.docx]

**Response to reviewer**

We thank the reviewer for their helpful comments, and have addressed each one below with our response shown in red.

Introduction

- The text included in this section is too long and imprecise. In this sense, the authors should only include the theoretical support related to the study. It is suggested that you review the following articles:

i. Thoits, P.A. Mechanisms linking social ties and support to physical and mental health. J. Health Soc. Behav. 2011, 52, 145–161. ,
ii. Uchino, B.N., et al. The relationship between social support and physiological processes: A review with emphasis on underlying mechanisms and implications for health. Psychol. Bull. 1996, 119, 488–531.,
iii. Uchino, B.N. Social support and health: A review of physiological processes potentially underlying links to disease outcomes. J. Behav. Med. 2006, 29, 377–387.,
iv. Smith, K.P. and Christakis, N.A. Social networks and health. Annu. Rev. Sociol. 2008, 34, 405–429

We have completely rewritten the introduction to ensure precision in language, and to not deviate from the topic at hand. We have also incorporated some of the suggested references as appropriate.

- It is not necessary to make the hypotheses explicit and the objective must be concise and precise.

We have removed the explicit hypothesis and rewritten this paragraph:

“*In this study, we build on previous research into stress, support and menopause symptoms by testing whether social support acts as an effective buffer against the negative influence of stress on VMS. First, we test whether higher levels of social support and stress associate with less frequent VMS and more frequent VMS, respectively. Further, we test whether social support buffers against the proposed negative impact of stress on VMS.”*

Methods

- What is the reliability of the instruments used to measure social support and stressful events?

We had already highlighted the reliability of the measure of stressful events (L293-295):
“*This is a modified version of the Psychiatric Epidemiology Research Interview life events scale (69), which has been validated across multiple racial and ethnic groups and shows high internal consistency (70).”*

A few sentences have now been added about the reliability of our social support measure (L283-286):

“*This measure is taken from the Medical Outcome Study: Social Support Survey (MOS-SSS) which is one of the most widely used instruments for measuring social support (69). The MOS-SSS has high levels of validity and reliability and has been translated into multiple languages (70-73).”*

Results

- The titles of the tables should be less long and more precise.
  Thank you for this suggestion. While recognising the value of concise titles, we have decided to keep the titles of the tables as they are. This is because we are aware that readers might just look at the tables for the results/quick summary of the paper (e.g., tables and graphs are frequently shared on social media or in talks without manuscript text). Therefore, we want to ensure the tables make sense in their own right, and we have deemed the titles of the tables necessary.
- You do not need to include the reference of the statistical analysis (this was already done in the methods section).

This has been removed.

Discussion

- The authors do not analyze or compare their results with similar studies. In this sense, you must consider the similarity of the instruments used to measure the variables

Thank you for this suggestion. We believe we have already done this in the following sentences:

- “*This is in line with findings that perceived stress is influential over symptomology (23), and that women who are deemed to be more resilient experience fewer menopause symptoms (31).*”
- “*Elsewhere in the literature, there are reports that various kinds of support (familial, emotional, etc.) are associated with fewer menopause symptoms (31, 33, 70), but we did not replicate these effects in our analysis. When looking at other domains of health, researchers have found that social support is not always protective against stress (71, 72). It may be in this case that the support question was not specific enough to capture its effect on menopause symptoms (e.g. women could have been asked “how often do you have someone to talk to about your health”),*”

Though, for clarity, we have added in the following sentence:

“*with previous research sometimes using combined measures of support in studies.*”

- The authors should consider that the self-report of stressful events are not necessarily related to the perception and response to stress.
  We are not completely clear what is meant by this comment, however, the measure of stress used in this study is self-reported and includes how upset the respondent is, which is surely subjective and therefore related to perception. For example, how upset someone is by a life stressor will be relative to past experience of stressful events, general resilience, etc. (Dohrenwend, 2006), and therefore we think that the measure of stress used in this study would be related to perception and response to stress.
- Study limitations should be included.
  Thank you for this suggestion. Limitations have been added in as follows:

“*This study is correlative, and therefore we cannot definitely say that stressful events found upsetting by a woman result in worse menopause symptoms. Further, the precise causal mechanism that might underly the relationship is not clear. Nonetheless, the persisting relationship between stress and menopause symptoms allows us to consider possible behavioural interventions women could use to deal with the symptoms associated with the menopausal transition.*”

Conclusion

- The conclusions are not based on the results

Thank you for highlighting this. The conclusion has been rewritten to remove any speculative conclusions, or where appropriate we have highlighted when they are speculative inferences of results.

References

DOHRENWEND, B. P. 2006. Inventorying stressful life events as risk factors for psychopathology: Toward resolution of the problem of intracategory variability. *Psychological Bulletin,* 132**,** 477-495.
