# Supplementary material for: The relationship between social support, stressful events, and menopause symptoms
Source: PLoS One. 2021 Jan 27;16(1):e0245444. doi: 10.1371/journal.pone.0245444 (PMC7840006; doi:10.1371/journal.pone.0245444)
Supplement: Attachment — Submitted filename: R2_Arnot et al. 2020_Reviewer reply.docx [file pone.0245444.s003.docx]

We would like to thank the reviewers for their helpful comments. We have addressed each one below, with our response shown in red.

Reviewer #1

- In general, the authors have made corrections to the manuscript considering the comments. However, I reiterate that it is necessary that the titles of tables 2 and 3 should be more concise. In this regard, the specifications can be included at the bottom of the table.
  Excess information has been moved to the bottom of the table.

Reviewer #2

- The authors state: “This research aims to clarify how support and stress are associated with one another in regards to VMS, as well as informing the potential efficacy of support-based treatments – such as mindfulness and mediation – to manage menopause symptoms.” This makes it sound like the authors are going to be looking at mindfulness and meditation (I think they meant this instead of “mediation”) in their analyses. However, in reading the rest of the manuscript, there is nothing about these concepts in the analyses. Also, this is the first mention of these constructs in the Introduction. I suggest that this sentence be removed and maybe moved to the Discussion as a future direction.
  This sentence has been removed from the introduction.
- With respect to the social support measure, I am concerned about the authors letting the data drive their analyses. In my reading, they first tested which social support measures best predicted VMS and then chose the one that best predicted VMS to go forward with in their analyses. First, this feels like data mining to me. Second, if social support is a moderator, there is no reason that it needs to be related to the outcome (see Baron & Kenny, 1986) – in other words, a moderator is supposed to be orthogonal to the predictor and outcome.
  Thank you for pointing this out. We were by no means trying to data mine. Rather, as it’s survey data with hundreds of possible variables, we were using pre-specified variable selection strategies. However, we have taken this comment on board and changed the measure of social support used in the analysis, and instead used a composite measure that includes the four forms of support collected by SWAN. This has not changed the overall results of the analysis, and we have included the individual measures in the S1 Text.
- Additionally, the authors highlight the race/ethnic differences in levels VMS symptoms and then speculate what that might mean. But, they have the data to conduct analyses to see if the link between stress and VMS differs by race/ethnicity and whether the support buffer is found for some race/ethnic groups as opposed to others.
  Thank you for highlighting this. We don’t wish to specifically add this as a research question as it is not one of our aims; however, we do now mention it more specifically in the results and comment on how our data fit the claims that stress underlies the relationship between ethnicity and VMS: “*Here, African American women experienced the most frequent symptoms, while Chinese and Japanese Americans reported symptoms less often, which is in line with findings from previous research (3, 9, 13, 75, 76). It has been suggested that the relationship between stress and menopause symptoms may help to explain some of this variation. In the US (where data used in this study was collected), African American individuals present more markers of stress than other ethnicities (77), likely due to economic and systematic factors (75). However, results in this study suggest that stress does not mediate the relationship between ethnicity and VMS, with the relationship persisting after the inclusion of the stress index and stress dose within the model (S1 Text Table C). Additionally, attributing ethnic differences to stress does not account for why women from an East Asian background in this sample report fewer symptoms, as – due to being an ethnic minority – it would be predicted that due to higher levels of stress (75) they would report more symptoms than white women. Therefore, it is unlikely that stress explains the ethnic differences in menopause symptoms, and further research is still required to understand this variation*.”
- Finally, they mention that a limitation of their analyses is that they are correlative. However, they have multiple waves of data and could easily have conducted lagged analyses to determine whether causation would be supported.
  Thank you for bringing this up. We do not necessarily think a lagged analysis would be theoretically appropriate: as we explain in the introduction, we are suggesting that current stress is driving the relationship between stress and VMS, and therefore we would likely need data on a shorter timescale to establish a definitive causal relationship. Further, by conducting a lagged analysis, we are still only really showing a correlation between stress at *t-1* and VMS, and so a significant effect could be found by chance. However, we have added in a *post hoc* analysis into the results section and discussion, were we find that current stress better predicts VMS than historic stress, which does support our notion that there is a causal relationship.
- In the first paragraph, first sentence, the authors say “The menopause is often a significant event for women…” I find the use of “The” to be awkward – I think it should just say “Menopause is often a significant event for women…”
  This has been changed.
- In Table 2, I think there is a typo – half the sample is Chinese American and only 7% white but that does not match with the text. Also, Caucasian is no longer used (it is actually attached to white supremacists: https://www.latimes.com/opinion/story/2019-09-10/race-caucasian-myth-racism) – instead, it should be listed as “White”.
  Thanks for pulling these issues up – they have been changed accordingly.
